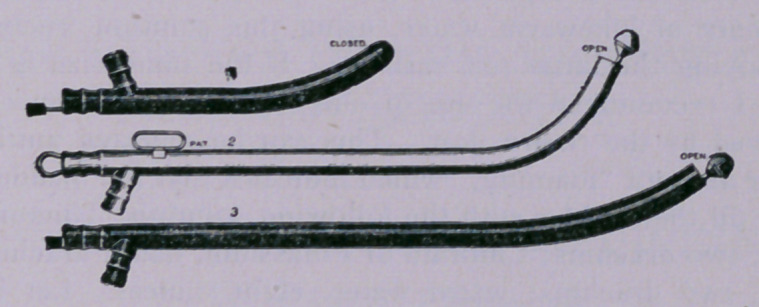# Notes and Notices

**Published:** 1899-02

**Authors:** 


### NOTES AND NOTICES.

### THE BACTERIOLOGICAL DIAGNOSIS OF DIPHTHERIA.

BALTIMORE, NOVEMBER 4th, 1898.

*To the Editor of the New York Medical Journal:*

SIR.—Now that we physicians of Baltimore are in the midst of quite a great deal of diphtheria, a recent experience of mine may be of interest to guard against putting too much confidence in an absolute diagnosis being made by bacteriological examination.

In one case in which a prompt recovery had been made in an infant it was desired to get certificates for two brothers to return to school. During the baby's illness one of the boys had had a sore throat and made a quick recovery; the other had not been sick at all. Cultures taken from both throats showed that the boy who had had the sore throat was free, while the case of the one who had not been sick at all was reported to me as being diphtheria. This occurred on October 19th, and up to date the boy has had no symptoms.

Another case was reported to me on the 29th ult. as not being diphtheria; clinically, however, it was the worst case I have ever seen. Two injections of two thousand units of antitoxine each were administered, but gave no relief, and intubation acted simply in prolonging life a few hours.

I am in no way attemping to cast ridicule on the bacteriological examination of cultures to determine a diagnosis. Errors could creep in and mistakes be made. I sent a second culture because of this, and it, too, was returned as negative.

We could be lulled into a sense of false security by a negative report, and such experiences as mine may help any statistics that are being collected on the treatment of this dread disease.

ALBERT S. ATKINSON, M. D.

(*New York Medical Journal.*)

A NEW ANÆSTHETIC.—Two Germans, investigators, MM. Einhorn and Heintz, have lately discovered a new anæsthetic which they call orthoform. It belongs to the group of aromatic amidoethers, and is a light crystalline white powder, tasteless, odorless, and of weak solubility. With acids it forms soluble salts, which are also anæsthetic, but too irritating to be employed locally on mucous membranes. Applied in powder or ointment to a wound or raw surface, orthoform renders them insensible —a fact confirmed by repeated clinical observation. In extensive burns, especially, orthoform allays the severest pains in a few minutes, and the relief endures for hours. Being non-poisonous, there is no danger in reapplying it as often as may be required after the first effect has ceased.

Thus, in a case of ulcerated cancer of the face, where constant and excruciating pain rendered sleep impossible orthoform to the amount altogether of fifty grams, was dusted over the sore for a whole week. Pain ceased, and no ill effect followed. The remedy is equally safe and effectual when administered internally as an anodyne in cancer of the stomach. Moreover, it is a powerful antiseptic, and consequently promotes healing. Orthoform has no effect on the unbroken skin, but owing to its decided action upon mucous membranes, may prove valuable as a local anæsthetic previous to operations on that region—a question whch is now experimentally determined at Munich.—*Paris Revue Scientifique.*

### TOOTING.

A tutor who tooted the flute,
Tried to teach two young tutors to toot;
Said the two to the tutor,
Is it harder to toot or
To tutor two tooters to toot?

See the picture in another column of lovely women in the Lagar; girls treading grapes to music at Quinto Cellieros, Portugal, wearing short jackets and pants. Speer, of New Jersey, has improved methods and the excellence of Speer's wines is attested by physicians throughout America and Europe who have used them. Orders are shipped to Dresden and Vienna.

MARCHAND'S PREPARATIONS.—When you cannot procure from your druggist my medicinal preparations in their original, unbroken package, viz., Peroxide of Hydrogen (medicinal), Glycozone, Hydrozone, and Eye Balsam, please address your order to my agent for Pennsylvania, Mr. Wm. T. Berry, 109 So. 11th Street, Philadelphia, who will fill same promptly.

Yours truly,

CHARLES MARCHAND,

Laboratory, 57-59 Prince Street Cor. New Elm Street, New York City.

DR. CYRUS EDSON of the New York Health Board, and Dr. Mott, of the Bellevue Hospital, give their unqualified endorsement to Speer's wines for the sick, and the debilitated and aged.

HOT WATER IN THE TREATMENT OF GONORRHŒA.—C. S. Murrell (*Massachusetts Medical Journal,* 1898, Vol. XVIII) advocates hot water irrigations in the treatment of acute and chronic gonorrhœa. The apparatus consists of a soft catheter, which is passed to within one inch of the prostatic urethra. It is then connected with a "gravity apparatus," in which the water is gradually heated up to the point of tolerance. The stream flows in through the catheter, and returns between the catheter and the mucous membrane. Several quarts of warm water may be used at each treatment. Some patients can tolerate a temperature as high as 180 or 190 degrees F. The following advantages are claimed for this method of treatment:

1. The course of the disease is shortened by at least two-thirds, making the average limit of the case—viz., stoppage of the discharge—nearer one week than three.

2. The discharge immediately changes from a purulent to that of the nature of gleet, and is reduced to a very small quantity.

3. There is absence of chordee and pain in passing urine.

4. Stricture, as a sequel, which is well understood to be the frequent result of using strong astringents, is improbable.—*Bacteriological Review.*

Serum-Therapy.—Geo. W. Cox, M. D. (*Journal of the American Medical Association,* Oct. 8, 1898), in a paper on this subject, gives an account of the history of serum-therapy and its value as a therapeutic measure.

The Turks are credited with being the first to use this form of treatment. Nearly two hundred years ago they practiced a crude form of inoculation against small-pox. Their methods were not practical, so the practice died out, and it was not until several decades later that Jenner revived the idea in the form of vaccination.

The practical application of serum-therapy was not made possible until Louis Pasteur discovered the relation of microbes to disease. Following this discovery, in rapid succession, the numerous and useful ideas now so familiar to every student of medicine, including the explanation of the cause and nature of infection, of contagion, and of the principles of prophylaxis and cure by vaccination.

Pasteur also gave to the world the new treatment for hydrophobia. Thus he laid the foundation upon which the superstructure of serum-therapy is now being erected. He is acknowledged by the whole world to be the "father of bacteriology" as it is known and practiced to-day. Through this relationship he must forever be designated as the *grand-father of serum-therapy.*

### CATHETERS AND CYSTITIS.

BY R. N. MAYFIELD, M. D., NEW YORK,

Formerly President of the Colorado State Board of Medical Examiners and lecturer in pathology and clinical medicine, University Colorado, etc.

It is well known that when it is necessary to use a catheter of usual construction—that is, with the ordinary fine perforations as an inlet thereto—it does not work readily or satisfactorily, or subserve fully the results expected from it.

Examples of such unsatisfactory operations are seen where there is a good deal of mucus present in the bladder, such mucus being apt to surround or lie upon the end of the catheter, clogging or stopping the apertures thereof and preventing the ingress of fluids to be drawn off; again, when sediment or calcareous matter is present, it clogs, even sometimes filling in part or completely the apertures, with consequent failure of the catheter to fully perform its functions. Such failures are especially apt to happen in nearly, if not quite, all forms of chronic diseases of the bladder, and notably so in cystitis.

My object, therefore, is to present a catheter that is reliable and efficient in operation when the use of a catheter is indicated in all conditions and diseases of the bladder. In this instrument the danger of clogging or failure to perform its functions is obviated, and its interior may be readily made aseptic, and bits of mucus that usually clog an ordinary catheter may be readily drawn off.

This catheter is of very simple construction, being tubular, with the curve of an ordinary instrument, and opened at the end for an inlet. For the closure of this open end, and for the easy insertion of the catheter, as well as for other purposes, a bulbous or rounded head is used, preferably solid, and attached to one end of a wire, passing through the body or tube and projecting at its rear outlet end.

This construction forms a very efficient catheter, having an area of opening so large as to greatly obviate the danger of clogging, for, if mucus should lodge against the open end, the working of the head back and forth upon its seat would cut away the obstructing bits of mucus and permit them to pass through the tube.

With this instrument there should be no hesitancy in using Nitrate of Silver, Iodine, Corrosive Sublimate, Carbolic-acid, or Hydrogen solutions in the bladder, as any of these solutions can be readily drawn off or neutralized, thus preventing poisoning from absorption, or preventing rupture from gases that form in the bladder.

Regarding the treatment of cystitis with the employment of this catheter, presuming that we have a typical case, with ropy, viscid, and tenacious mucus, the membrane thickened and possibly ulcerated, and in deep folds "ribbed," as it were we begin the treatment as follows:

1. Inject a quarter of a grain of Cocaine dissolved in a drachm of water into the membraneous portion of the urethra.

2. Anoint the largest hard-rubber catheter that can be well pressed into the bladder, and increase the size one number each week until the urethra is normal in size.

3. Begin with dilute Hydrogen solutions—preferably Hydrozone—one part to twenty of lukewarm water, using this solution freely, especially when employing the large size catheter. If the small size is used at the beginning, I recommend the use of only two or three ounces at a time until removed by the return flow. This can be repeated until the return flow is clear and not "foaming," which indicates that the bladder is aseptic.

4. Partly fill the bladder with the following solution: Tincture of Iodine Compound, two drachms; Chlorate of Potassium, half a drachm; Chloride of Sodium, two drachms; warm water, eight ounces. Let it remain a minute or so and then remove. This treatment should be used once or twice a day.

Where I suspect extensive ulceration I recommend once a week the use of from ten to twenty grains of Nitrate of Silver to the ounce, and neutralize with Chloride of Sodium solutions.

This treatment carried out carefully will be satisfactory, as there is no remedy that will destroy bacteria, fœtid mucus, or sacculated calcareous deposits like Hydrogen.—*New York Medical Journal,* September 3d, 1898.

## Figures and Tables

**Figure f1:**